# A22 USING AN AIR-LIQUID INTERFACE (ALI) COLONOID MODEL TO DEFINE INTESTINAL MICROBE-MUCUS INTERACTIONS: CITROBACTER RODENTIUM EMPLOYS ESPC TO PENETRATE MOUSE COLONIC MUCUS

**DOI:** 10.1093/jcag/gwae059.022

**Published:** 2025-02-10

**Authors:** Y Chen, Q Liang, A Gilliland, H Yang, H Yu, B vallance

**Affiliations:** Pediatric, The University of British Columbia, Vancouver, BC, Canada; Pediatric, The University of British Columbia, Vancouver, BC, Canada; Pediatric, The University of British Columbia, Vancouver, BC, Canada; Pediatric, The University of British Columbia, Vancouver, BC, Canada; The University of Kansas Medical Center, Kansas City, KS; Pediatric, The University of British Columbia, Vancouver, BC, Canada

## Abstract

NOT PUBLISHED AT THE AUTHOR’S REQUEST

**Funding Agencies**: None

